# Parental Exposure to Nanoplastics and Graphene Oxide Induces Transgenerational Toxicity in *Caenorhabditis elegans*

**DOI:** 10.1002/jat.70250

**Published:** 2026-05-29

**Authors:** Gabriela Corrêa Soares, Larissa Müller, Suamy Machado Cruz, Andy Taipe Huisa, Danilo Roberto Carvalho Ferreira, Clascídia Aparecida Furtado, Juliane Ventura‐Lima

**Affiliations:** ^1^ Universidade Federal do Rio Grande – FURG Rio Grande Rio Grande do Sul Brazil; ^2^ Programa de Pós‐Graduação em Ciências Fisiológicas – Instituto de Ciências Biológicas (ICB) – Universidade Federal do Rio Grande – FURG Rio Grande Rio Grande do Sul Brazil; ^3^ Centro de Desenvolvimento da Tecnologia Nuclear – CDTN Belo Horizonte Minas Gerais Brazil

**Keywords:** emerging contaminants, nanoparticles, oxidative stress, polystyrene, reproduction

## Abstract

Environmental pollution by emerging contaminants such as nanoplastics (NPs) and graphene oxide (GO) represents a growing threat to global health and ecosystems. This study investigated the effects of parental exposure, both isolated and combined, to 100 μg/L NPs (polystyrene, spherical, 100 nm) and 100 mg/L GO, using *Caenorhabditis elegans* as a biological model. Parental exposure to these contaminants induced oxidative stress and alterations in antioxidant defense enzymes (SOD, CAT, GST), leading to lipid peroxidation and physiological impairments such as reduced reproduction, locomotion, and growth. Notably, these biochemical disruptions were also evident in unexposed offspring, indicating transgenerational inheritance of toxic effects. Overall, our findings highlight persistent oxidative damage as a central mechanism underlying NPs‐ and GO‐induced toxicity and emphasize the need to incorporate generational studies into environmental risk assessment to better predict long‐term impacts of emerging contaminants.

## Abstract 2

This study investigated the effects of parental exposure, both isolated and combined, to nanoplastics (100 μg/L) and graphene oxide (100 mg/L) using *Caenorhabditis elegans* . Exposure induced oxidative stress and caused reproductive and locomotor impairments. These effects were also observed in unexposed offspring, indicating transgenerational toxicity. Overall, our findings highlight oxidative damage as a central mechanism and emphasize the importance of transgenerational approaches in environmental risk assessment.

## 1 Introduction

Environmental pollution is currently one of the major threats to the integrity of ecosystems and global public health, associated with negative impacts on biodiversity and human well‐being. Rapid population growth, urban expansion, and industrial advancement have contributed to the introduction of a wide variety of essential products but also the insertion of chemical compounds into the environment (Manzetti and van der Spoel 2015). Among these several contaminants, there are those called emerging, which are chemical substances that are not traditionally monitored but have the potential to cause adverse effects on the environment and human health due to their persistence, bioaccumulation, and toxicity (Xingyu et al. 2024). Given their complexity and the possibility of inducing persistent effects, investigating the toxicity mechanisms of emerging contaminants, in isolated and/or combined exposures, is fundamental for understanding their ecotoxicological risks and for developing mitigation strategies. Among the emerging contaminants, we can highlight nanoplastics (NP) and graphene oxide (GO).

NPs are plastic particles with at least one dimension measuring 100 nm or less (Liang et al. 2021; León et al. 2024), originating both from the fragmentation of larger plastics and from direct manufacturing for use in products such as cosmetics and personal care items. These particles have a high surface area, high chemical reactivity, the ability to adsorb toxic compounds, and the potential to cross biological barriers (Lai et al. 2022). These properties give NPs the ability to interact directly with living organisms, affecting cellular and physiological processes such as the induction of oxidative stress, inflammation, and changes in cell viability (Qian et al. 2025).

In parallel, the advancement in the development and application of nanomaterials, such as GO, has raised important questions about their environmental and toxicological impacts. GO, a functionalized form of graphene containing oxygenated groups, is widely used in biomedicine, electronics (Parvin et al. 2024), and environmental remediation (Gill et al. 2023). Furthermore, GO is incorporated as an additive in various polymer materials, enhancing mechanical and thermal stability (Liu et al. 2022; Cong et al. 2018). Similar to NPs, GO exhibits a high surface area, facilitating cellular internalization and conferring toxic potential. Although GO has been extensively studied in isolation, the potential effects arising from combined exposure with other emerging contaminants, particularly NPs, remain largely unexplored, highlighting a critical gap in environmental risk assessment.

Co‐exposure to different nanomaterials in the environment is increasingly reported, although its ecotoxicological implications remain poorly understood. Graphene‐based materials have been widely investigated as adsorbents for environmental remediation (Liu et al. 2022), which may contribute to their presence in contaminated systems. In addition, graphene derivatives are incorporated into polymer materials, potentially leading to the co‐occurrence of both NPs and GO (Liu et al. 2022). Interactions between NPs and GO may result in synergistic or antagonistic effects, influencing bioavailability, aggregation behavior, and toxicity (Biswas and Sarkar 2019). Previous studies, including those from our group, have shown that exposure to microplastics and other nanomaterials can induce multigenerational effects in model organisms (Soares et al. 2023; Josende, Nunes, Müller, dos Santos Francisco, et al. 2019). However, the potential for combined exposure to NPs and GO to induce persistent or transgenerational effects remains poorly understood.

In this context, multigenerational research has gained prominence by investigating the long‐term effects of environmental pollutants (Nacci et al. 2002; Guédon et al. 2021). These studies are crucial for understanding the consequences of prolonged exposure on organisms and ecosystems, given the persistence of many contaminants in nature. Multigenerational toxicity refers to adverse effects observed across successive generations that were directly exposed to the contaminant, whereas transgenerational toxicity describes effects manifested in generations that have never had direct contact with the pollutant, indicating heritable transmission of toxic outcomes (Zhao et al. 2017).

Several model organisms are used in generational studies, but only a few can join the necessary characteristics with the capability of dwelling both in soil and aquatic environments (Gubert et al. 2023). The nematode *Caenorhabditis elegans* ( *C. elegans* ) stands out as an experimental model for multigenerational and transgenerational toxicology studies due to its short life cycle, high sensitivity to low concentrations of environmental pollutants, ease of genetic manipulation, and high fertility (Gonzales‐Moragas et al. 2015; Müller et al. 2023). These characteristics allow for rapid and detailed observation of the effects of different pollutants over several generations, making *C. elegans* a powerful tool to investigate the molecular and genetic mechanisms underlying inherited toxicity.

Based on these characteristics, several studies have shown that exposure to pollutants such as organic compounds, metals, and nanomaterials in the parental generation has been shown to cause toxic effects in subsequent generations that were not directly exposed. The main effects observed include developmental toxicity (with reduced body size), decreased locomotion, reproductive toxicity (such as reduced number of offspring), as well as oxidative stress and epigenetic changes that can be inherited and contribute to the persistence of toxic effects (Jin et al. 2022., Liu et al. 2021, Zhao et al. 2017).

Therefore, the objective of the present study was to evaluate, both in isolation and co‐exposure, the two emerging contaminants of environmental concern, NPs and GO, in the free‐living nematode *C. elegans* , using a transgenerational approach, to elucidate the potential toxic effects not only on the exposed generation but also on subsequent ones. In this way, we seek to understand the ecotoxicological risks associated with the simultaneous presence of these nanoparticles, to contribute to mitigation strategies and ecosystem protection.

## 2 Materials and Methods

### 2.1 NPs and GO Characterizations, Suspension Preparation and Experimental Concentrations

Subsequently, we present information about the contaminants. For spherical polystyrene nanoparticles (PS‐NPs; diameter, 100 nm) were purchased from Sigma‐Aldrich (LB1; lot number MKCT5832) and spherical fluorescent polystyrene nanoparticles (FRPS‐NPs; diameter, 100 nm), exclusively used for NPs accumulation, were also purchased from Sigma‐Aldrich (L9904; lot number MKCW3102). Physicochemical properties from PS‐NPs and FRPS‐NPs are given as follows: scanning electron microscopy (SEM) utilizing the JEOL JSM 6610 microscopy (Figure S1 and Figure S2), zeta potential and dynamic light scattering (Table S1) are presented as supplementary materials. To avoid agglomeration and improve dispersion, the liquid PS‐NPs and FRPS‐NPs suspension used in the experiments was prepared with EPA water and sonicated (Eco‐sonics Q3.0/40A, 40 kHz, 110 W) for 30 min before each generational exposure.

The GO was obtained by oxidizing natural graphite, following the original method developed by Hummers and Offeman (1958) with modifications by Marcano et al. (2010). Natural graphite was supplied by the Brazilian company, Nacional de Grafite Ltda. A 1.65‐g/L GO stock solution prepared in EPA water was used for the exposures. Before each exposure, the bottle containing the GO stock solution was placed in an ultrasonic washer (Ultrasonic Cleaning Soni‐tech, Sonifier 450 digital, 20 kHz, 400 W) for 5 min at 10% amplitude to avoid GO aggregation. To evaluate purity, structural quality, oxidation degree, and morphology, the GO sample was characterized by dynamic light scattering and zeta potential (Table S2), Raman spectroscopy (Figure S3), Fourier transform infrared spectroscopy (FTIR; Figure S4), acid–base potentiometric titration (Table S3), UV–Vis absorbance (Figure S5), and X‐ray diffraction (XRD; Figure S6).

The concentrations used in this study (100 μg/L of PS‐NPs/FRPS‐NPs and 100 mg/L of GO) were selected based on previous studies showing biochemical and physiological effects of these nanomaterials when tested individually (Wu et al. 2013; Sun et al. 2021). These concentrations are known to induce measurable, yet sublethal, responses, allowing the assessment of toxicity at different biological levels. The aim of this study was not to reproduce environmental concentrations or establish a dose–response relationship, but to explore potential mechanisms of toxicity.

Although the concentrations differ by three orders of magnitude, this reflects differences in the physicochemical properties and biological potency of each nanomaterial, as reported in previous studies. Each concentration was selected based on its ability to induce measurable, yet sublethal responses when tested individually. In this context, the combined exposure was designed to investigate potential interactions under conditions where both materials are biologically active.

### 2.2 *C. elegans*

The wild strain N2 Bristol *C. elegans* was cultivated in the nematode growth medium (NGM) containing 17 g/L bacteriological agar, 3 g/L NaCl, 2.5 g/L peptone, 25 mL of 0.5 M KH_2_PO_4_ buffer (pH 6.0), 1 mL of 1 M MgSO_4_, 1 mL of 1 M CaCl_2_, 1 mL of 0.005 g/L cholesterol dissolved in ethanol, and 1 mL antibiotic/antimycotic solution (100×; Sigma‐Aldrich). The Petri dishes for nematode cultivation were filled with NGM to two‐thirds of their volume and seeded with *Escherichia coli* bacteria ( *E. coli* OP50; nonpathogenic strain) as a food source for the nematodes at an optical density of 2.8 (600 nm). Prior to the experiment, the animals in the culture plates were synchronized by filtration method by a 28‐μm mesh to restrict the population to the L1 stage. This filtering synchronization was chosen to avoid any possible chemical interference in the findings. After this procedure, the obtained L1 larval stage animals were placed in Petri dishes containing NGM until hatching, after which *E. coli* OP50 was added. The Petri dishes were placed in an incubator at 20°C for another 96 h until the experiment started.

### 2.3 Transgenerational Assay

Transgenerational assessments evaluate the effects of previous exposed generations driven across unexposed generations. Thus, on the day of exposure, the nematodes present in the culture plates were washed using EPA water [CaSO_4_.2H_2_O (60 mg/L), MgSO_4_ (60 mg/L), KCl (4 mg/L) and NaHCO_3_ (96 mg/L)] to carry out the collection of the animals. The resulting suspension underwent a physical synchronization process through filtration in a 28‐μm mesh. After synchronization, the population density was quantified by employing the circular estimation method (CEM), a more accurate and precise method developed in our laboratory (Josende, Nunes, Müller, Cravo, et al. 2019). Approximately 10,000 L1 larvae were transferred to each Petri dish (with a 100‐mm diameter model K13‐Kasvi), with three plates being allocated to each experimental group as follows: control group (animals without contact with contaminants, fed with *E. coli* OP50 in EPA water), NPs group (animals exposed to PS‐NP at a concentration of 100 μg/L + *E. coli* OP50 in EPA water), GO group (animals exposed to GO at a concentration of 100 mg/L + *E. coli* OP50 in EPA water), and NPs‐GO group (animals subjected to simultaneous exposure to PS‐NP at 100 μg/L and GO at 100 mg/L + *E. coli* OP50 in EPA water).

The initial exposure occurred in NGM medium containing a thin layer of EPA water (1.65 mL), seeking to simulate environmental conditions closer to natural ones (Soares et al. 2023). The L1 larvae of the parental generation (F0) were immediately exposed to the specific treatments of each group and maintained for a period of 96 h. At the end of this period, individuals were collected and used to obtain the F1 generation, from which there was no further contact with the contaminants.

The experiment extended until the fourth generation (F3). In each generational transition, the animals from each plate were collected in Falcon tubes containing 50 mL EPA water, where three 250‐μL samples were collected for growth and reproduction analysis and one 1‐mL sample was collected for behavior analysis. The L1 larval population was separated by filtration, quantified again through the CEM method, and approximately 10,000 individuals were transferred to initiate the subsequent generation. Finally, the remaining adult animals were divided into five parts for the biochemical analyses. The entire experiment was conducted in triplicate.

### 2.4 NP Accumulation Qualitative Assay

To detect NPs in the bodies of animals from F0 utilizing FRPS‐NPs, they were anesthetized with 10 μL of NaN3 (20 μmol) and inspected by epifluorescence microscopy, using the standard fluorescein filter set (excitation 480 nm, emission 525 nm, respectively). The epifluorescence microscopy was performed using an Olympus IX81 inverted epifluorescence microscope. Digital images were captured with an Olympus DP72 CCD camera and analyzed with FIJI free software.

### 2.5 Biochemical Analysis

#### 2.5.1 Sample Preparation

The nematode suspension samples in EPA water liquid medium at 1:1 volume were kept on ice and homogenized by sonication (QSonica, model Q125; power, 50% amplitude, 125 W) for 40 s. After this process, the samples were centrifuged at 10,000×*g* for 20 min at 4°C. For biochemical assays, three independent biological replicates were used per treatment in each generation, each corresponding to animals collected from a separate culture plate. The protein content in each collected supernatant was quantified in a microplate reader at 600 nm using a commercial kit (Microprote Pyragollol; Bioclin) based on the Bradford method. Finally, the samples were stored in an ultra‐freezer at −80°C for further biochemical analysis as described below.

#### 2.5.2 Superoxide Dismutase Activity

To evaluate the activity of superoxide dismutase (SOD) enzyme, we used the methodology developed by McCord and Fridovich (1969), where the xanthine oxidase system generates the superoxide radical anion (O_2_^●−^), and the reduction of cytochrome C is measured at 550 nm with a spectrophotometer (BioMate 3, Thermo Scientific). The SOD activity was expressed in SOD units, defined as the amount of enzyme required to inhibit 50% of the reduction of cytochrome C per minute per milligram of protein at 25°C and pH 7.8.

#### 2.5.3 Catalase Activity

To determine catalase (CAT) enzyme activity, the methodology described by Beutler et al. 1975, where decomposition of 50 mM of H_2_O_2_ was quantified at 240 nm for each sample. The results obtained were expressed as CAT units, where 1 unit corresponds to the hydrolysis of 1 μmol of H_2_O_2_/min and per milligram of protein at 25°C and pH 8.0.

#### 2.5.4 Glutathione S‐Transferase Activity

To evaluate the glutathione S‐transferase (GST) enzyme activity, we followed the method determined by Habig and Jakoby 1981. The method is based on the generation of a complex at 340 nm by conjugating 1 mM reduced glutathione (GSH) with 1 mM 1‐chloro‐2,4‐dinitrobenzene (CDNB; Sigma‐Aldrich), which was detected using a spectrophotometer (ELx 800, BioTek), and the results were expressed as nmol/CDNB conjugated per minute per milligram of protein.

#### 2.5.5 Levels of Thiobarbituric Acid Reactive Substances

To evaluate lipid peroxidation, the method described by Oakes and Van Der Kraak (2003), where lipid peroxide levels were assessed through the reaction of malondialdehyde (MDA) with thiobarbituric acid (TBA, JT Baker). This reaction occurs under acidic pH and elevated temperature conditions, forming a chromogen that can be quantified by fluorimetry (Victor 2, PerkinElmer microplate reader). The excitation and emission wavelengths of 520 nm and 580 nm were applied, respectively. Tetramethoxypropane (TMP, Sigma‐Aldrich) was used as the standard. Results were expressed as nanomoles of thiobarbituric acid reactive substances (TBARS) per milligram of protein.

### 2.6 Physiological Analysis

#### 2.6.1 Growth

At the end of each generation, three samples composed of 10 animals each were randomly collected from those three 250‐μL samples mentioned in Section 2.4 at every new generation (96 h). The growth of the animals was determined by measuring the body length. The measurements were performed using the free software ImageJ. Results were expressed as growth length (mm) (Josende, Nunes, Müller, et al. 2019).

#### 2.6.2 Reproduction

At the end of each generation, reproduction assessment was estimated by CEM (Josende, Nunes, Müller, Cravo, et al. 2019), where three samples of each one of those three 250‐μL aliquots mentioned in Section 2.4 were estimated. The results were expressed as the number of offspring per fertile adult, as described by Josende, Nunes, Müller, et al. (2019).

#### 2.6.3 Behavior

At the end of each generation, a 1‐mL sample of animals per plate were collected for behavioral analysis. The animals were placed into a 15‐mL Falcon tube and washed with 13 mL EPA water to remove bacterial content. After washing, each Falcon tube was left undisturbed for 5 min to form a pellet. Then, the supernatant was removed until 2 mL was left per tube. This procedure was carried out three times, and at the third instance, the supernatant was removed until only 100 μL remained. The pellet was transferred to a 1.5‐mL Eppendorf tube and gently resuspended by tube inversion to ensure an even distribution of animals. Then, 50 μL was transferred to the well of a 24‐well plate. Subsequently, 300 μL of EPA water was added to form a thin layer at the bottom of the well. The animals were allowed to recover for 1 min before being recorded for 1 min for the body bending assay using a Leica microscope (model DFC295) and OBS Studio software (version 27.2.4, 64‐bit). Worm movement was recorded for 60 s, and the videos were converted to slow motion for analysis. The number of body bends was counted, with each bend defined as a complete change in bending direction involving the whole body, observed at the mid‐body region. Locomotion was quantified manually through video analysis following standardized criteria. The analyses were not performed under blinded conditions. The recorded videos were independently evaluated by trained observers, each analyzing a subset of videos. All treatments were assessed within each analytical set, and data were compiled after completion of the evaluations. Each treatment in each generation was conducted using three independent biological replicates. Within each replicate, five animals were randomly selected and analyzed for locomotion. Data were expressed as the number of complete swimming movements per animal.

### 2.7 Statistical Analysis

Statistical data were tested for normality and homogeneity of variances using Cochran's and Levene's tests, respectively, and mathematical transformations were applied when necessary. The experimental design consisted of three independent plates, each containing all treatments (control, NPs, OG, and NPs + OG). Within each plate, five animals were analyzed per treatment, resulting in a total of *n* = 15 animals per treatment. This design follows a standardized protocol adopted in previous studies using *C. elegans* under controlled laboratory conditions. All parameters were analyzed using two‐way analysis of variance (ANOVA), considering treatment and generation as factors, followed by the Newman–Keuls test for multiple comparisons. The level of significance was set at 5% (*α* = 0.05), according to Zar (1984).

## 3 Results

This study evaluated the results of three treatment groups: NPs (100 μg/L), GO (100 mg/L), and combined NPs + GO (100 μg/L + 100 mg/L) and a control group across four generations. To assay the transgenerational effects of the treatment groups, only the first generation (F0) was directly exposed.

### 3.1 NP Accumulation

Nanoplastics (FRPS‐NPs) were observed in the animal intestine (adults and young adults) from the parental generation (F0) from NPs and NPs + GO groups. Epifluorescence microscopy revealed detectable fluorescence in nematodes exposed to NPs (100 μg/L); a continuous fluorescent signal was observed along the intestine, indicating widespread internal distribution. In contrast, animals co‐exposed to NPs and GO (100 μg/L + 100 mg/L) showed a distinct pattern of discrete NPs agglomerates within the intestinal tract, suggesting that GO alters NPs distribution or aggregation in vivo (Figure 1).

**FIGURE 1:**
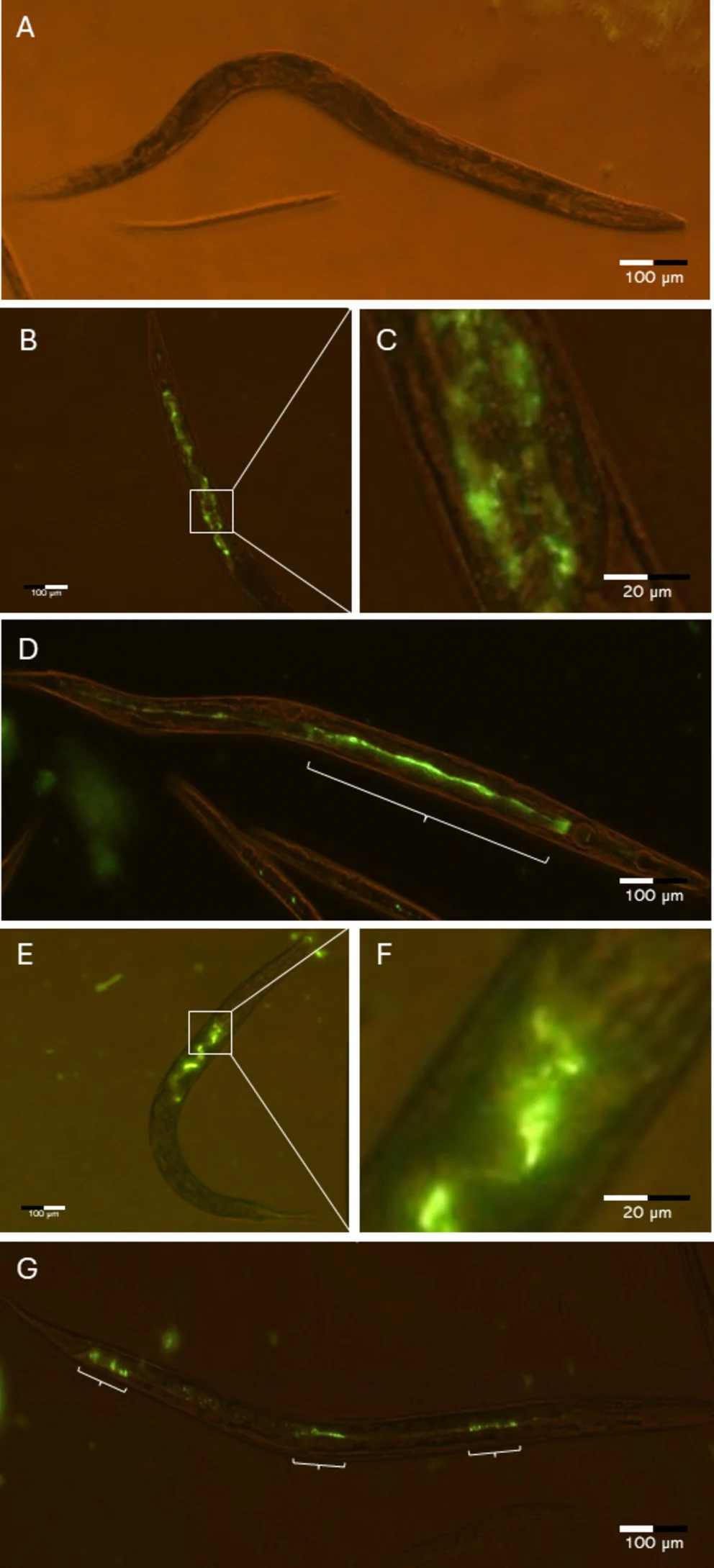
NP accumulation. In vivo NPs accumulation: *C. elegans* images obtained by epifluorescence microscopy. (A) Animal from the CTL group under fluorescent field (100× magnification); (B) adult animal from the NPs (100 μg/L) group under fluorescent field (60× magnification); (C) inset of the selected area from panel B showing a continuous green NPs distribution pattern within the *C. elegans* intestine; (D) young adult from the NPs (100 μg/L) group under fluorescent field (100× magnification), showing a continuous green NPS distribution pattern within the intestine; (E) adult animal from the NPs + GO (100 μg/L + 100 mg/L) group under fluorescent field (60× magnification); (F) inset of the selected area from panel E showing a green NPs agglomerate pattern within the *C. elegans* intestine; (G) young adult from the NPs + GO (100 μg/L + 100 mg/L) group under fluorescent field (100× magnification), showing agglomerated green NPs within the intestine. Scale bars = XX μm.

### 3.2 Biochemical Responses

#### 3.2.1 SOD

In F1, SOD enzyme activity was significantly increased in the NPs and NPs + GO groups compared to the CTL (*p* < 0.05). In F2, a similar increase was observed in the NPs and GO groups when compared to the CTL (*p* < 0.05; Figure 2).

**FIGURE 2:**
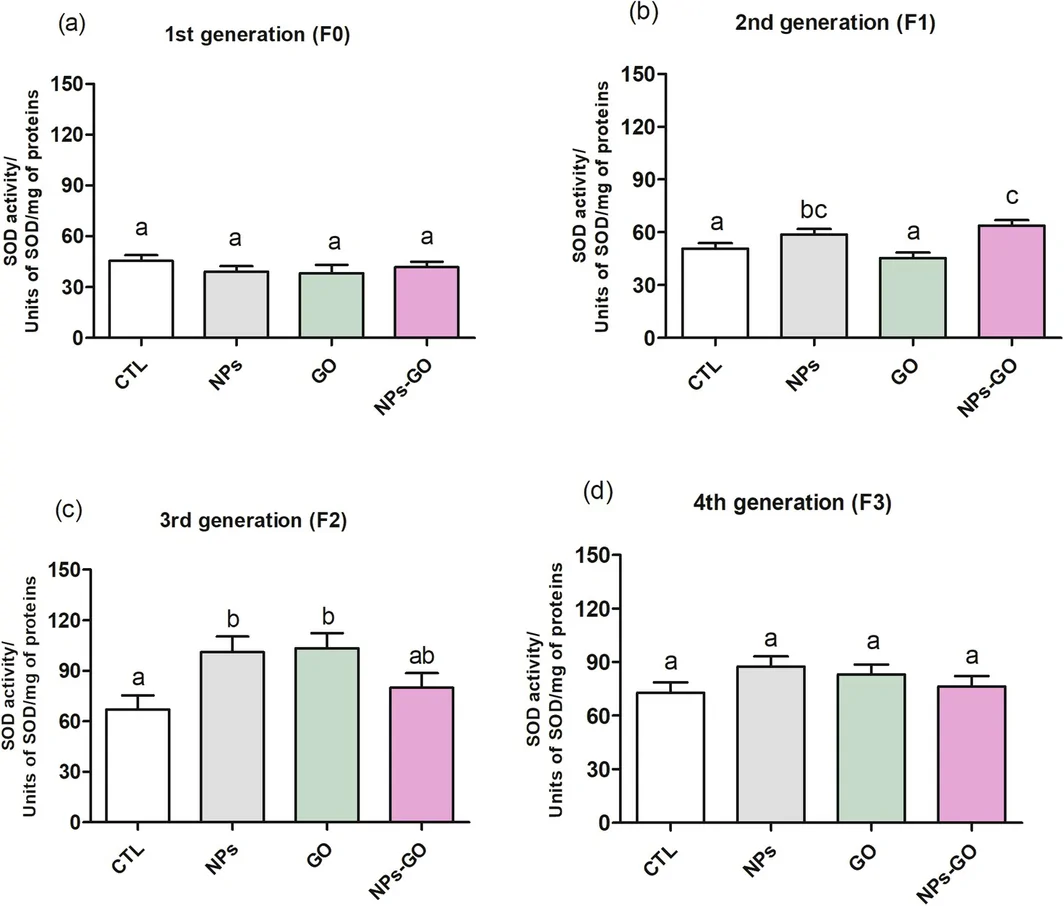
SOD activity across four generations (F0–F3). Values are shown as mean ± SEM (*n* = 15 measurements derived from three independent biological replicates). Different letters indicate significant differences among groups (*p* < 0.05), while shared letters indicate no significant difference.

#### 3.2.2 CAT

In F1 and F2, CAT activity was significantly increased in the NPs, GO, and NPs + GO groups compared to the CTL (*p* < 0.05). In F3, only the NPs group showed a significant increase compared to the CTL (*p* < 0.05; Figure 3).

**FIGURE 3:**
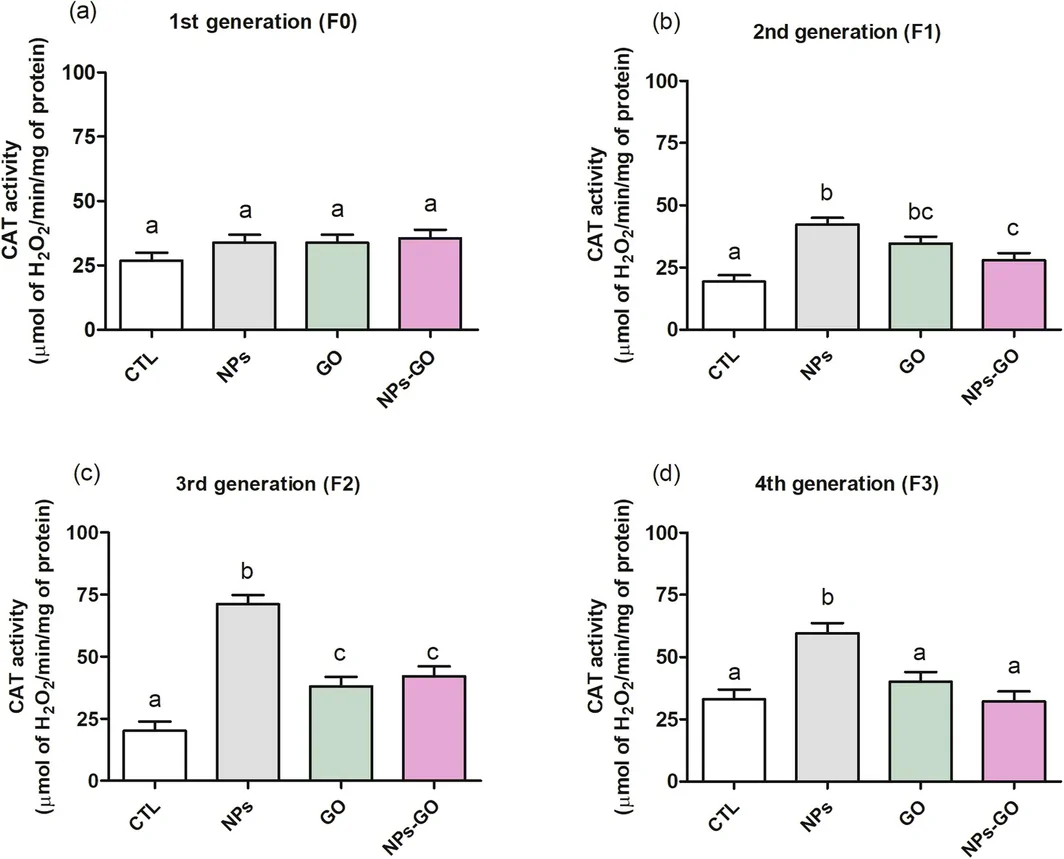
CAT activity across four generations (F0–F3). Values are shown as mean ± SEM (*n* = 15 measurements derived from three independent biological replicates). Different letters indicate significant differences among groups (*p* < 0.05), while shared letters indicate no significant difference.

#### 3.2.3 GST

In F0, GST activity was significantly increased only in the NPs group compared to the CTL (*p* < 0.05). In F1, a significant increase was observed exclusively in the NPs + GO group (*p* < 0.05). In F2, GST activity was significantly elevated in the NPs and GO groups compared to the CTL (*p* < 0.05). In contrast, in F3, the NPs group showed a significant increase in GST activity, while the GO and NPs + GO groups exhibited a significant decrease compared to the CTL (*p* < 0.05; Figure 4).

**FIGURE 4:**
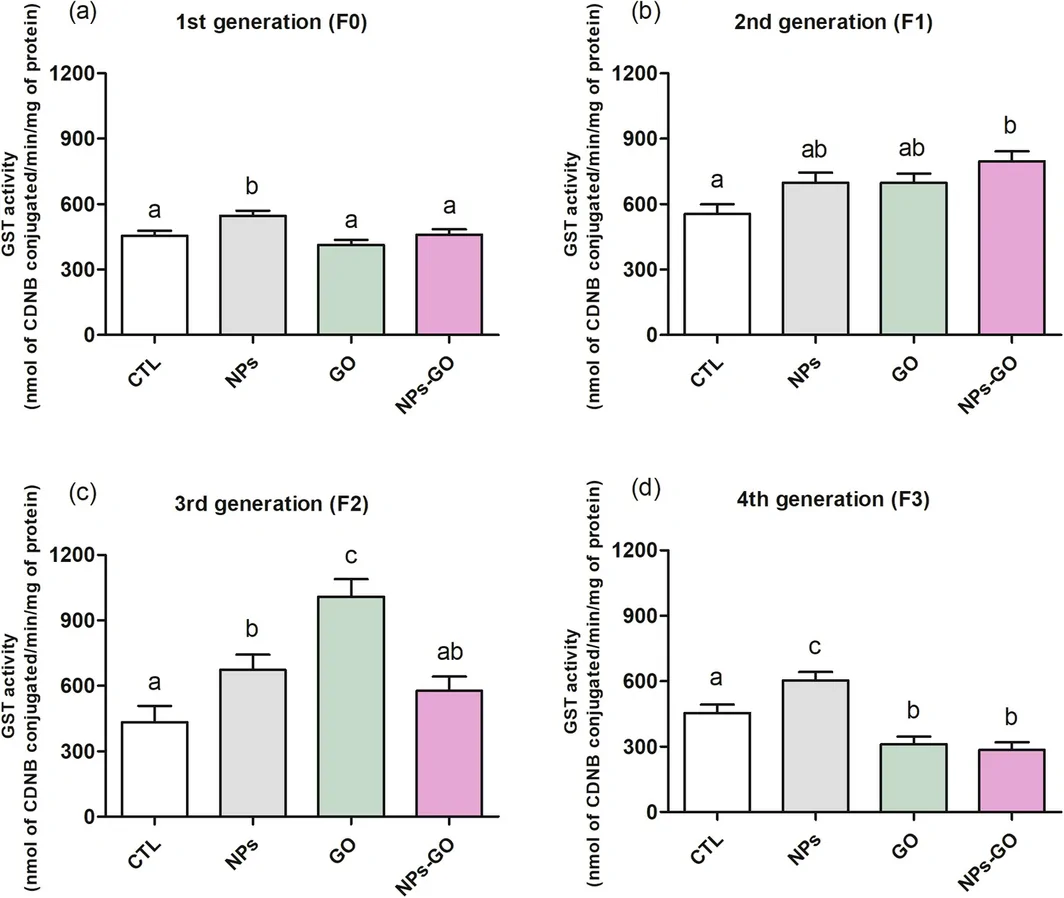
GST activity across four generations (F0–F3). Values are shown as mean ± SEM (*n* = 15 measurements derived from three independent biological replicates). Different letters indicate significant differences among groups (*p* < 0.05), while shared letters indicate no significant difference.

#### 3.2.4 TBARS

In F0, lipid peroxidation levels were significantly increased only in the NPs + GO group compared to the CTL (*p* < 0.05). In F1, TBARS levels were significantly elevated in both the NPs and NPs + GO groups (*p* < 0.05). In F2, increased lipid peroxidation was observed in the GO and NPs + GO groups compared to the CTL (*p* < 0.05; Figure 5).

**FIGURE 5:**
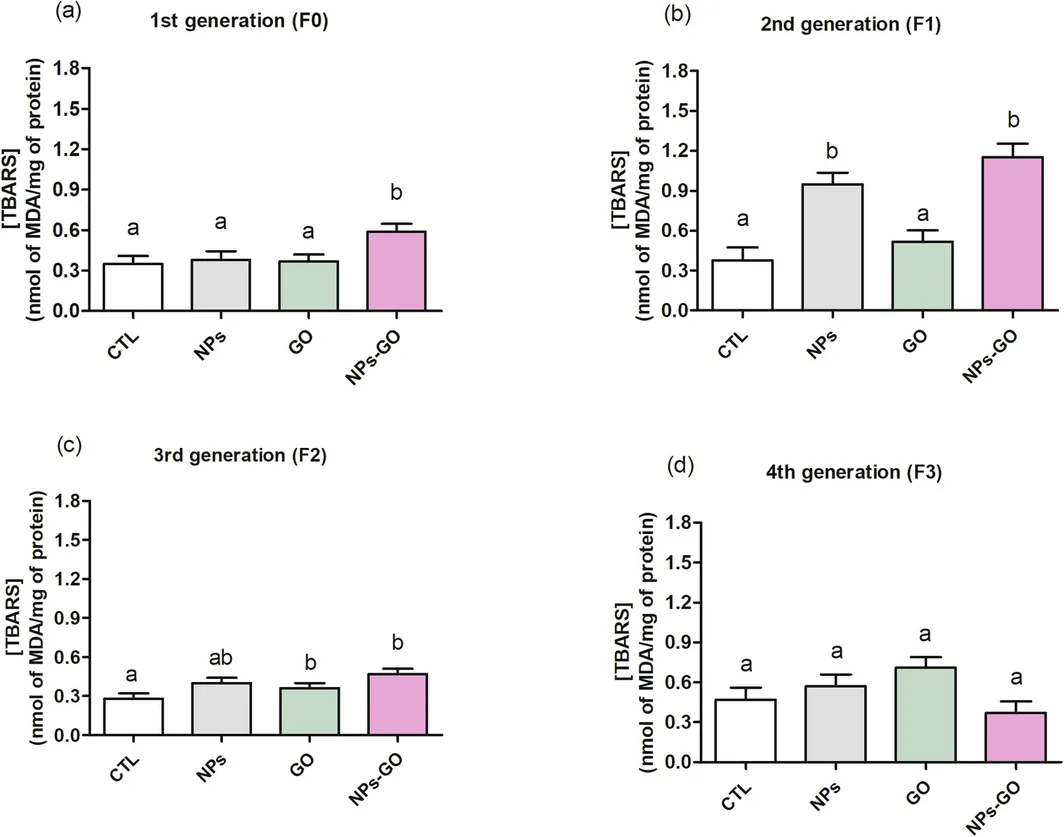
Lipid peroxidation levels (TBARS) across four generations (F0–F3). Values are shown as mean ± SEM (*n* = 15 measurements derived from three independent biological replicates). Different letters indicate significant differences among groups (*p* < 0.05), while shared letters indicate no significant difference.

### 3.3 Physiological Responses

#### 3.3.1 Body Bending

A significant decrease in body bends was observed in GO and NPs + GO animals in F0 compared to the CTL (*p* < 0.05). In F2, all groups showed impaired locomotory capacity relative to the CTL (*p* < 0.05). No significant differences in locomotion were observed in F1 and F3 when compared to the CTL group (Figure 6).

**FIGURE 6:**
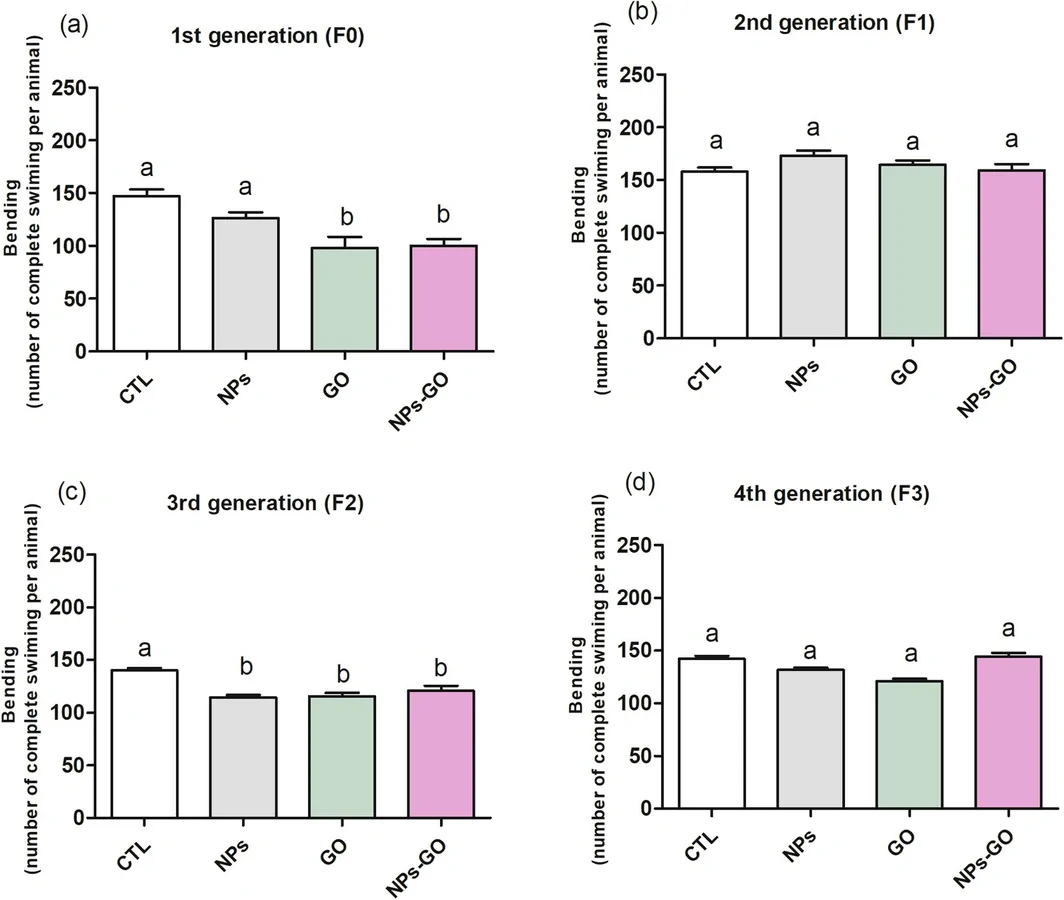
Body bending across four generations (F0–F3). Values are shown as mean ± SEM [*n* = 15 animals from three independent biological replicates (five per replicate)]. Different letters indicate significant differences among groups (*p* < 0.05), while shared letters indicate no significant difference.

#### 3.3.2 Growth

Overall, individuals in the NPs, GO, and NPs + GO groups showed a significant decrease in body size compared to the CTL group (*p* < 0.05). This reduction was consistent across all four generations (Figure 7).

**FIGURE 7:**
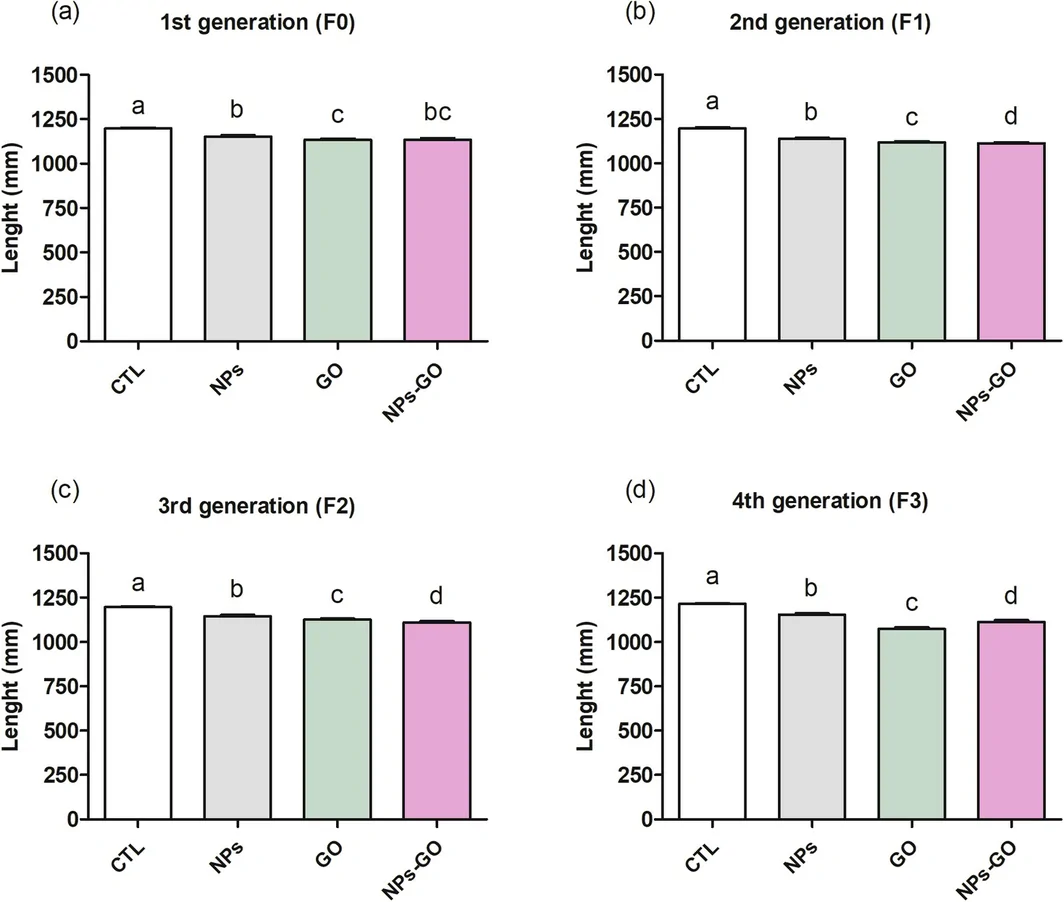
Growth of adult individuals across four generations (F0–F3). Values are shown as mean ± SEM [*n* = 15 animals from three independent biological replicates (five per replicate)]. Different letters indicate significant differences among groups (*p* < 0.05), while shared letters indicate no significant difference.

#### 3.3.3 Reproduction

A significant decrease in reproduction was observed in the NPs and NPs + GO groups in F0 compared to the CTL (*p* < 0.05). In F1, no significant differences were detected among the groups. In F2, reproduction was significantly reduced in the GO and NPs + GO groups (*p* < 0.05), while in F3, this reduction was observed in all treated groups compared to the CTL (*p* < 0.05; Figure 8).

**FIGURE 8:**
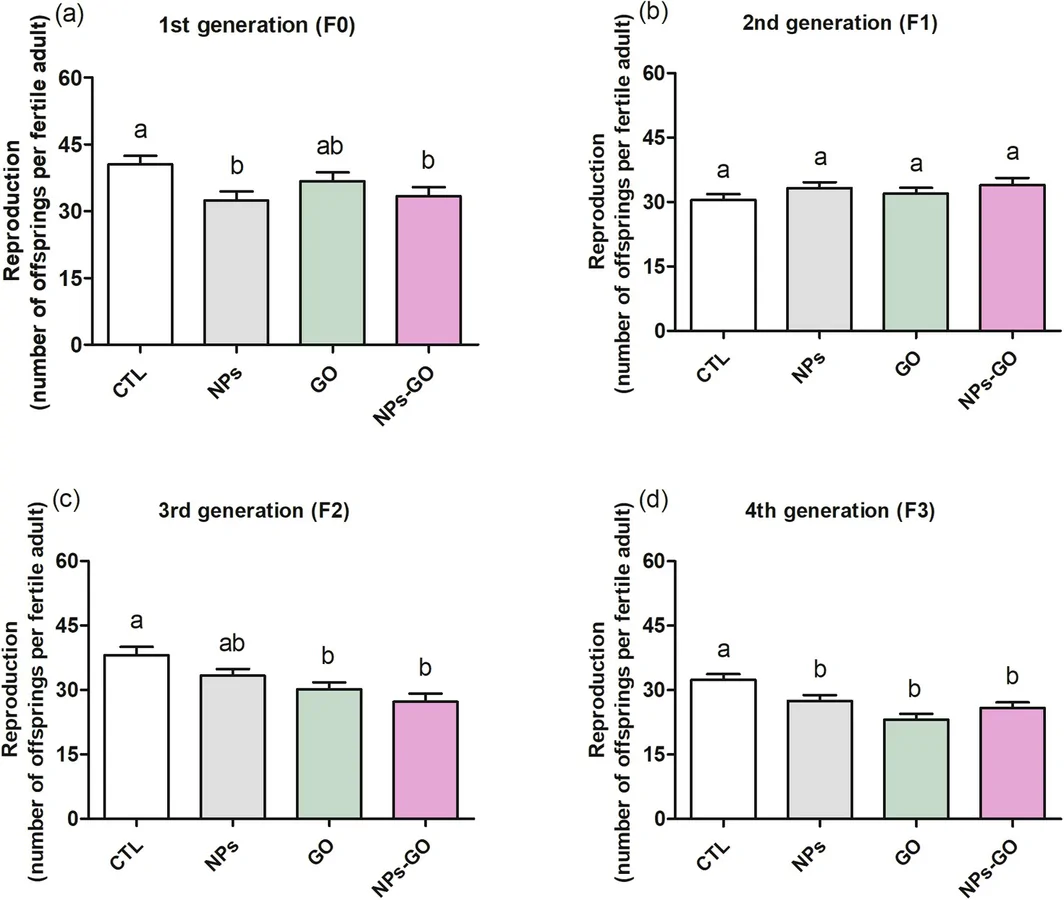
Reproduction across four generations (F0–F3). Values are shown as mean ± SEM [*n* = 15 animals from three independent biological replicates (five per replicate)]. Different letters indicate significant differences among groups (*p* < 0.05), while shared letters indicate no significant difference.

## 4 Discussion

In the present study, we investigate the biochemical and physiological toxic effects of the sole exposure of a parental generation of *C. elegans* to NPs and GO separately, as well as in combination (NPs + GO). The effects were monitored in the sampled generation and along with three subsequent unexposed generations, therefore performing a proper transgenerational study.

### 4.1 NP Accumulation

In the first step of this study, parental generation (F0) animals from NPs and NPs + GO groups were scanned by epifluorescence microscopy to detect whether the exposed animals (adults and young adults) have the capacity of accumulating NPs into their bodies. Our study confirmed the NPs accumulation into the bodies of animals from the parental generation (F0) exposed to the NPs group (Figure 1b—adult; 1d—young adult) preferably spread along the intestine, and the NPs + GO group (Figure 1e—adult; 1 g—young adult) preferably agglomerated in the intestine. This observation reveals that sampled animals had the capacity to intake the NPs in a 96‐h exposure (F0). Despite the accumulation pattern, the presence of GO in the co‐exposure group (NPs + GO) appears to influence the distribution of NPs, leading to a greater accumulation in the *C. elegans* intestine. A previous multigenerational study showed that *C. elegans* exposed to 5 and 50 μg/L spherical polystyrene (1.1 μm) microplastics presented the same capacity of intake and accumulation into different sections of the organisms, mostly inside the foregut, midgut, and hindgut (Soares et al. 2023). Yu et al. (2021) also observed NPs accumulation in the intestine of *C. elegans* in the F0 generation while in subsequent generations (F1–F4) the presence of NPs was not detected. Qian et al. (2025) also observed the NPs accumulation in the intestine of *C. elegans* . These studies show that the intestine is the target organ for NPs accumulation in this nematode and can induce several effects because this organ is responsible for coping with toxic and other chemical stressors of the environment.

The detection of NPs in animals from the F0 generation confirms that organisms inevitably interact with contaminants present in their environment. The observation of toxic effects in unexposed offspring indicates that exposure during the parental generation was sufficient to induce long‐lasting biological alterations. These findings demonstrate that, although NPs were not physically transferred to the next generations, their residence time in the parental generation was enough to trigger transgenerational toxic effects.

### 4.2 Biochemical Effects

The organisms possess a complex antioxidant defense system (ADS) responsible for protection against reactive oxygen species (ROS), pro‐oxidant agents, and other contaminants (Sies and Jones 2020). In the present study, the activities of SOD (Figure 2), CAT (Figure 3), and GST (Figure 4) were evaluated, along with lipid peroxidation as an indicator of oxidative damage (Figure 5). Although specific studies on the combined effects of GO and NPs are still limited, our results did not indicate a clear additive or synergistic effect. This pattern is consistent with previous studies that, despite focusing on molecular endpoints, also report responses without clear interaction effects between contaminants.

Previous studies have demonstrated that exposure to GO or NPs can induce oxidative stress responses in nematodes. Wu et al. (2013) reported increased intestinal ROS production and upregulation of SOD‐2 and SOD‐3 genes following exposure to GO (0.5–100 mg/L). Similarly, exposure to 100 nm PS‐NPs (1–10 μg/L) resulted in increased ROS levels and altered SOD‐3 expression (Qiu et al. 2020). Moreover, co‐exposure to PS‐50 (1 μg/L) and PS‐500 (15 μg/L) enhanced the expression of genes related to oxidative stress responses, including CAT (ctl‐3), SOD‐2, and SOD‐3 (Wu et al. 2023).

SOD and CAT act directly on ROS, catalyzing the dismutation of superoxide anion and the decomposition of hydrogen peroxide, respectively, and thus represent key components of the antioxidant defense system. In this study, both enzymes showed a similar response pattern, with no significant changes observed in the directly exposed generation (F0) (Figure 2a and Figure 3a). In contrast, increased enzyme activity in subsequent generations suggests that the effects of exposure are not restricted to directly exposed organisms, but may persist across generations, possibly reflecting inherited physiological adjustments following parental exposure.

GST is an enzyme that catalyzes the conjugation of reduced glutathione (GSH) with electrophilic toxic compounds, including lipid peroxidation products. This reaction enhances their aqueous solubility and facilitates excretion, thereby contributing to the detoxification of electrophiles and mitigating cellular damage induced by oxidative by‐products. The increase in GST activity observed in the parental generation (F0) (Figure 4a) in the NPs‐exposed group may represent an immediate response to eliminate ROS or toxic lipid peroxidation products. However, the variation in GST activity observed across subsequent generations suggests a regulated adjustment of detoxification processes over time. Importantly, when comparing single and combined exposures, GST activity did not show a consistent increase under co‐exposure conditions. Instead, responses varied according to treatment and generation, with increases observed in NPs or GO groups individually rather than consistently in the NPs + GO group. This indicates that co‐exposure does not produce additive or synergistic effects on GST activity but rather reflects distinct modes of action of each nanomaterial on detoxification pathways.

When comparing our findings with previous studies, León et al. (2024) reported that exposure of *C. elegans* to 10,000 ng/L of PS‐NPs increased the expression of the *gst‐4* gene. Similarly, Qu et al. (2023) observed that NPs exposure upregulated *nduf‐7* and *nuo‐6*, which encode subunits of mitochondrial complex I. Such upregulation may lead to mitochondrial dysfunction, resulting in enhanced ROS production and activation of antioxidant defenses. Together, these findings support the role of NPs exposure in modulating redox homeostasis. In our study, in the parental generation (F0), the absence of significant changes in antioxidant enzymes such as SOD and CAT does not necessarily indicate the absence of a pro‐oxidative condition under NPs exposure. The observed NPs accumulation, together with other physiological alterations, supports the presence of a harmful state. Although ROS were not directly measured, the activities of SOD and CAT, which use superoxide anion and hydrogen peroxide as substrates, respectively, provide indirect evidence of changes in oxidative status. It is possible that other antioxidant mechanisms not evaluated in this study were sufficient to counteract the ROS generated in the parental generation. However, GST showed a distinct response, with increased activity in F0 (Figure 4a) and variation across subsequent generations, indicating a specific involvement of this enzyme in the detoxification response to nanomaterial exposure.

Interestingly, in groups exposed only to NPs, the increase in GST activity was not accompanied by lipid peroxidation, supporting a protective role of this enzyme. In contrast, under co‐exposure to GO, distinct patterns were observed, with increased GST activity in F1 (Figure 4b) and decreased activity in F3 (Figure 4d). Despite these variations, co‐exposure was associated with increased lipid peroxidation in specific generations (Figure 5a–c). However, these increases were not consistently higher than those observed under single exposures, suggesting that co‐exposure does not necessarily intensify oxidative damage.

Overall, the increased activities of antioxidant enzymes (CAT, SOD, and GST), particularly in subsequent generations, indicate a sustained physiological response to oxidative challenges induced by nanomaterial exposure. At the same time, the occurrence of lipid peroxidation demonstrates that these defenses were not sufficient to fully prevent oxidative damage under either single or combined exposure conditions.

In the present study, the comparison between single and combined exposures did not reveal a consistent increase in toxicity under co‐exposure conditions. Instead, the responses varied depending on the endpoint and generation, with combined exposure often resulting in effects comparable to those observed for NPs or GO alone. This pattern suggests that interactions between these nanomaterials may modulate biological responses, rather than simply enhancing toxic effects.

A possible explanation for these patterns, although still speculative, may involve epigenetic‐related mechanisms triggered by ancestral exposure, such as germline‐mediated regulatory processes that could influence the expression of oxidative stress defense genes, including SOD, CAT, and GST. Although these mechanisms were not directly assessed in this study, this hypothesis is supported by the modulation of antioxidant enzyme activities observed across generations under both single and combined exposures, suggesting that the responses detected in later generations may reflect inherited adjustments in redox regulation following exposure to NPs and GO, either individually or in combination.

Although this response may have a protective role, it could also become maladaptive if it disrupts redox balance or imposes unnecessary metabolic costs. In this context, our findings suggest that ancestral exposure to toxicants can influence the physiological responses of subsequent generations, even in the absence of continued exposure.

Previous studies provide support for this interpretation. For instance, Yu et al. (2021) reported that NPs exposure induced germline apoptosis associated with increased ROS levels in non‐exposed generations of *C. elegans* (F1–F4), whereas no such effects were observed in the F0 generation. Based on these findings, it is possible that NPs exposure (alone or combined with GO) created a pro‐oxidative condition in F0 in our study. However, because ROS levels were not directly measured, this interpretation should be considered with caution. It is also possible that redox balance was maintained by antioxidant mechanisms not evaluated here, although these responses may not have been sufficient to fully prevent oxidative damage, particularly in more sensitive cell types such as germline cells.

Similarly, Sun et al. (2021) showed that parental exposure to 100 μg/L NPs reduced germline cell numbers in F1 and F2, indicating reproductive toxicity that gradually declined in later generations. They also observed a peak of germline apoptosis in F1, suggesting that the activation of apoptotic pathways during transgenerational transmission may play an important role in these effects, with partial recovery occurring in subsequent generations. Although germline integrity was not directly assessed in this study, it is plausible based on these previous reports that NPs‐induced oxidative stress may affect germline cells. One possible mechanism involves lipid peroxidation, which can reduce membrane fluidity, increase permeability (e.g., ion leakage and organelle disruption), and damage mitochondrial membranes, ultimately leading to apoptosis or necrosis (Fueser et al. 2022). In this context, the oxidative alterations observed here, reflected by changes in antioxidant enzyme activity and lipid peroxidation, may be associated with modifications in germline function, potentially contributing to the effects observed across generations. However, these mechanisms remain speculative and require direct investigation to be confirmed.

The generational pattern of biochemical responses does not indicate a strictly progressive development of oxidative stress, but rather a dynamic response that varies across generations and exposure conditions. In the directly exposed generation (F0), the limited and selective changes observed suggest an initial but relatively constrained response. In F1, the increase in antioxidant enzyme activities, together with lipid peroxidation in some treatments, indicates an early compensatory response aimed at maintaining redox balance. In F2, the broader changes across multiple biomarkers suggest a shift in oxidative responses, indicating that compensatory mechanisms may not be sufficient under certain conditions. By F3, the absence of lipid peroxidation, along with the persistence of enzymatic changes in specific treatments, points to adaptive adjustments over generations, possibly reflecting a reorganization of redox regulation.

Biochemical alterations were detected across generations, but responses varied among biomarkers and exposure periods, indicating that transgenerational effects did not follow a uniform pattern. Instead, the observed changes suggest complex modulation of oxidative responses over successive generations. Similar variabilities in multigenerational responses have been reported in previous studies (Yu et al. 2021; Sun et al. 2021).

### 4.3 Physiological Effects

Physiological responses are among the most important toxicological endpoints due to their significant consequences for both individual organisms and populations. Although growth and reproduction are widely recognized as primary biological functions, a range of physiological biomarkers plays a key role in mediating these processes. Among them, behavior stands out as it is closely associated with both growth and reproduction, serving as a sensitive indicator of physiological status and environmental conditions. This parameter can ultimately determine the success or failure of individuals, populations, and even colonies (Qu et al. 2019). For example, locomotion (aquatic movement) is one of the most commonly assessed behavioral indicators of organism health, and its impairment can reduce survival and compromise population continuity.

In this study, we observed that F0 animals exposed to GO and NPs + GO showed a reduction in locomotion, as indicated by decreased swimming movements (Figure 6a), suggesting an impairment in locomotory capacity. In contrast, no significant differences were observed in the F1 generation compared to the CTL group. However, this impairment reappeared in the F2 generation (Figures 6b and c), while locomotory capacity was restored in the F3 generation (Figure 6d). These findings are in agreement with previous studies of León et al. (2024), which reported that exposure to 10,000 ng/L of aged PS‐NPs reduced locomotion in *C. elegans* , as assessed by body bends. Similarly, Wu et al. (2014) observed reduced locomotor activity in nematodes exposed to 100 mg/L of GO.

In the present study, although locomotion was restored by the F3 generation, the reductions observed in F0 and F2 suggest transient physiological impairment, possibly associated with stress responses induced by nanomaterial exposure. Notably, these effects were more evident in groups exposed to GO and NPs + GO in F0, indicating that GO, alone or in combination with NPs, may contribute to short‐term alterations in locomotory behavior. From a functional perspective, impairments in locomotion may affect essential activities such as foraging and environmental exploration, potentially compromising individual performance. Although these effects were transient, they indicate that nanomaterial exposure can temporarily interfere with organismal behavior.

Another important physiological biomarker is growth, as individuals showing impairment in this parameter tend to be less successful in reproduction. Moreover, reduced body size may increase susceptibility to predation by other organisms. In the present study, we observed a consistent decrease in growth in all treated groups compared to the CTL group across all assessed generations (Figure 7). Similar findings have been reported in the literature. Yang et al. (2015) observed reduced growth in *C. elegans* exposed to 100 mg/L of GO, while Yu et al. (2020) reported a 20% decrease in growth in organisms exposed to 100 μg/L of 1 μm spherical polystyrene MP. Together, these findings, along with the present study, provide strong evidence that both NPs and GO, either individually or in co‐exposure, can negatively affect growth.

From a physiological perspective, the consistent reduction in growth observed across generations may reflect increased energetic demands associated with stress responses, including the activation of antioxidant and detoxification pathways. This reallocation of energy from growth to maintenance processes may compromise overall organismal performance, even in the absence of clear differences between single and combined exposures.

Finally, reproduction is considered one of the most important physiological biomarkers, as negative effects on this parameter can have significant consequences for population survival. It is widely recognized in *C. elegans* studies as a sensitive indicator of physiological health, aging, and responses to environmental stress (Pickett et al. 2013; Josende, Nunes, Müller, dos Santos Francisco, et al. 2019). In the present study, all treatments, except in the F1 generation, negatively affected the reproduction of *C. elegans* (Figure 8). Similar findings have been reported in the literature. Wu et al. (2014) observed that exposure to 100 mg/L of GO in the parental generation (F0) reduced reproductive capacity by approximately 10% in the F1 wild‐type N2 strain. In addition, mutant nematodes were more susceptible to the same GO exposure, showing reductions ranging from 15% to 30% depending on the type of mutation.

Previous studies have demonstrated transgenerational effects of NPs on reproduction in *C. elegans* . Sun et al. (2021) reported that exposure to 100 μg/L of 35 nm NPs in the parental generation induced reduced reproduction in subsequent unexposed generations, associated with a decrease in the number of fertilized eggs. Similarly, Yu et al. (2021) observed transgenerational reproductive effects following exposure to 100 mg/L of NPs for 72 h. In addition, Sun et al. (2021) described a decline in reproductive capacity in the exposed F0 generation, followed by a gradual recovery in later generations (F3 and F4), possibly related to germline apoptosis induced by the particles. Despite the limited number of studies addressing co‐exposure to GO and NPs across generations, our results demonstrate that a single parental exposure to both nanomaterials can induce adverse effects in the offspring of *C. elegans* , even in generations not directly exposed. Notably, the transient recovery observed in F1 (Figure 8b), followed by a decline in F2 and F3 (Figures 8c and d), contrasts with the gradual recovery pattern reported by Sun et al. (2021). This difference suggests distinct dynamics in transgenerational reproductive toxicity. The pattern observed in this study may be associated with alterations in germline function, potentially linked to processes such as oxidative damage and germline apoptosis, as suggested in previous studies. The transient recovery observed in F1 appears insufficient to protect subsequent generations, reinforcing the potential role of transgenerational mechanisms, including heritable epigenetic changes, in affecting the viability of germline cells in unexposed descendants.

## 5 Conclusion

The magnitude of these transgenerational effects can be influenced by factors such as pollutant concentration, the developmental stage at which exposure occurs, duration of exposure, particle size and chemical properties, as well as mechanisms common among species, such as maternal transfer and epigenetic alterations. Findings in *C. elegans* demonstrate that environmental toxicity can extend beyond the directly exposed generation, affecting descendants through complex mechanisms probably involving genetic and epigenetic changes. This study expands the understanding of environmental risks and reinforces the need to consider long‐term effects in the assessment of the safety of environmental contaminants. Taken together, the transgenerational effects observed on oxidative stress biomarkers, behavior, and reproductive parameters in *C. elegans* indicate that exposure to NPs and GO, even if restricted to the parental generation, may have lasting biological consequences. These findings emphasize the need to include transgenerational and mechanistic endpoints in ecotoxicological assessments, given their potential to affect population viability and ecosystem‐level processes.

## Funding

Juliane Ventura‐Lima received a productivity research fellowship from Conselho Nacional de Desenvolvimento Científico e Tecnológico (CNPq) (process number 312359/2023‐3). This study was financed by Fundação de Amparo à Pesquisa do Estado do Rio Grande do Sul (FAPERGS) (process number 21/2551‐0001968‐9) granted to Juliane Ventura‐Lima, PhD.

## Ethics Statement

The authors have nothing to report.

## Conflicts of Interest

The authors declare no conflicts of interest.

## Consent to Participate

All authors consent to participate.

## Consent to Publish

All authors consent to publication.

## Supporting information


**Table S1:** Zeta potential and dynamic light scattering (DLS): (A) PS‐NPs and (B) FRP‐NPS.
**Table S2:** Zeta potential and dynamic light scattering (DLS) GO.
**Table S3:** Potentiometric titration was used to identify and quantify the functional groups present in the GO sample. The obtained pKa values were associated with different functional groups: carboxylic acids (pKa 4.0 and 5.8), lactones (pKa 7.0), and phenols (pKa 8.8 and 9.7; Konkena et al. 2012; Serodre et al. 2019), which are commonly found in GrO and GO samples synthesized by similar methodologies. The determined amounts were 12.69 mmol g⁻1 for carboxylic groups, 0.89 mmol g⁻1 for lactones, and 3.74 mmol g⁻1 for phenols, highlighting a predominance of –COOH groups on the nanostructure's surface.
**Figure S1:** Image SEM PS‐NPs.
**Figure S2:** Image SEM FRPS‐NPs.
**Figure S3:** The Raman spectrum exhibits the characteristic bands of graphitic structures: the G band, centered at 1582 cm⁻1, is associated with the stretching of C=C bonds in the hexagonal lattice, reflecting regions with order and crystalline integrity; the D band, centered at 1353 cm⁻1, is related to the breathing mode of carbon rings, activated by the presence of defects in the graphene hexagonal lattice (Cançado et al. 2007; Marcano et al. 2010; Saleem et al. 2018). The intensity ratio between the D and the G bands (ID/IG) close to 1.1 indicates a significant presence of defects, attributed to the introduction of oxygen‐containing groups into the graphitic lattice.
**Figure S4:** The FTIR spectrum shows characteristic absorption bands of GO, including –OH stretching of alcohols and phenols (3400 cm⁻1), –OH stretching of carboxylic acids (3230 cm⁻1), C=O stretching (1720 cm⁻1), C=C stretching from the graphitic domains (1625 cm⁻1), C–O stretching of phenolic groups (1427 cm⁻1), and C–O stretching of epoxy groups (1010 and 900 cm⁻1), confirming the presence of various oxygen‐containing functional groups introduced during the oxidation of graphite (Serodre et al., 2019; Surekha et al. 2020; Botella et al. 2022).
**Figure S5:** The presented graph shows the absorbance spectrum of a GO, which displays a characteristic profile for this material, with a single prominent peak around 230 nm, attributed to the π → π* transition of aromatic C=C bonds in the graphitic regions. The relatively high absorbance intensity suggests good colloidal dispersion of the GO and a significant concentration of oxidized sheets. The continuous decrease in absorbance across the spectrum, extending into the near‐infrared (NIR) region, is associated with n → π* transitions of C=O groups and scattering effects from partially reduced or aggregated sheets. This spectral behavior indicates the presence of oxygen‐containing functional groups, suggesting the material is still oxidized, although partially reduced with some conjugated domains. However, the absence of a new peak near 300 nm confirms that the material remains predominantly in its oxidized form (GO), rather than being reduced graphene oxide (rGO) (Serodre et al. 2019; Marcano et al. 2010).
**Figure S6:** XRD was used to characterize the conversion of natural graphite (expanded and milled) into graphite oxide (GrO). The obtained diffractogram shows a peak around 10°, corresponding to the (002) reflection of graphite oxide, and the absence of the peak at 26.5°, which is typical of the (002) reflection of natural graphite (expanded and milled), indicating the effective conversion of graphite into GrO (Pisapia et al. 2009; Stobinski et al. 2014; Saleem et al. 2018). Based on Bragg's Law, an interlayer spacing of 8.0 Å was calculated for GrO. This increase compared to the spacing of expanded graphite (3.4 Å) is attributed to steric and electrostatic repulsion effects caused by the oxygen‐containing groups introduced onto the surface of the layers, which weaken the π–π interactions between the graphitic planes.

## Data Availability

The data that support the findings of this study are available from the corresponding author upon reasonable request.
