# Supplementary material for: Parental Exposure to Nanoplastics and Graphene Oxide Induces Transgenerational Toxicity in Caenorhabditis elegans
Source: J Appl Toxicol. 2026 May 29;46(12):4826–40. doi: 10.1002/jat.70250 (PMC13727362; doi:10.1002/jat.70250)
Supplement: Supplementary file 1 [file JAT-46-4826-s001.docx]

**Supplementary information**

**Parental Exposure to Nanoplastics and Graphene Oxide Induces Transgenerational Toxicity in *Caenorhabditis elegans*.**

Gabriela Corrêa Soares^ab^, Larissa Müller^ab^, Suamy Machado Cruz^ab^, Andy Taipe Huisa^ab^, Clascídia Aparecida Furtado^c^, Juliane Ventura-Lima^ab^

^a^Instituto de Ciências Biológicas (ICB), Universidade Federal do Rio Grande - FURG, Rio Grande, RS, Brazil

^b^Programa de Pós-Graduação em Ciências Fisiológicas – FURG, Brazil

^c^ Centro de Desenvolvimento da Tecnologia Nuclear – CDTN, Brazil

**Corresponding author**

Ventura-Lima, J. e-mail: [juliane_ventura@yahoo.com.br](mailto:juliane_ventura@yahoo.com.br) or [ventura.julianelima@gmail.com](mailto:ventura.julianelima@gmail.com) - Universidade Federal do Rio Grande – FURG, Instituto de Ciências Biológicas – ICB. Avenida Itália, Km 08, Rio Grande - RS, CEP: 96203-900, Brazil.

**Fig.S1** Image scanning electron microscopy (SEM) PS-NPs

**Fig.S2** Image scanning eletron microscopy (SEM) FRPS-NPs

| **A Zeta potential** | | **Dynamic light scttering** | |
| --- | --- | --- | --- |
| Distribution peak (mV) | Mean (mV) | Polydispersity index [%] | Mean (nm) |
| **-24,4** | **-23,8** | **24,2** | **0,33** |
|  |  |  |  |
| **B Zeta potential** | | **Dynamic light scttering** | |
| Distribution peak (mV) | Mean (mV) | Polydispersity index [%] | Mean (nm) |
| **-16,7** | **-16,8** | **21,9** | **0,34** |
|  |  |  |  |

**Table S1** Zeta Potential and dynamic light scattering (DLS): (**A**) PS-NPs and (**B**) FRP-NPS

| **Zeta potential** | | **Dynamic light scttering** | |
| --- | --- | --- | --- |
| Distribution peak (mV) | Mean (mV) | Polydispersity index [%] | Mean (nm) |
| **-49,4** | **-45,1** | **16,9** | **692,3** |
|  |  |  |  |

**Table S2** Zeta Potential and dynamic light scattering (DLS) graphene oxide (GO)

**Fig.S3** The Raman spectrum exhibits the characteristic bands of graphitic structures: the G band, centered at 1582 cm⁻¹, is associated with the stretching of C=C bonds in the hexagonal lattice, reflecting regions with order and crystalline integrity; the D band, centered at 1353 cm⁻¹, is related to the breathing mode of carbon rings, activated by the presence of defects in the graphene hexagonal lattice (Cançado et al., 2007; Marcano et al., 2010; Saleem et al., 2018). The intensity ratio between the D and G bands (I_D_/I_G_) close to 1.1 indicates a significant presence of defects, attributed to the introduction of oxygen-containing groups into the graphitic lattice.

**Fig.S4** The FTIR spectrum shows characteristic absorption bands of graphene oxide, including –OH stretching of alcohols and phenols (3400 cm⁻¹), –OH stretching of carboxylic acids (3230 cm⁻¹), C=O stretching (1720 cm⁻¹), C=C stretching from the graphitic domains (1625 cm⁻¹), C–O stretching of phenolic groups (1427 cm⁻¹), and C–O stretching of epoxy groups (1010 and 900 cm⁻¹), confirming the presence of various oxygen-containing functional groups introduced during the oxidation of graphite. (Serodre et al., 2019; Surekha et al., 2020; Botella et al., 2022).

| **Functional group** | **pKa** | **Concentration (mmol g⁻¹)** |
| --- | --- | --- |
| Carboxyl | 2.0–6.0 | 12.69 |
| Lactone | 6.0–7.0 | 0.89 |
| Phenol | 8.0–10.0 | 3.74 |
| Total |  | 17.32 |

**Table S3.** Potentiometric titration was used to identify and quantify the functional groups present in the graphene oxide (GO) sample. The obtained pKa values were associated with different functional groups: carboxylic acids (pKa 4.0 and 5.8), lactones (pKa 7.0), and phenols (pKa 8.8 and 9.7) (Konkena et al., 2012; Serodre et al., 2019), which are commonly found in GrO and GO samples synthesized by similar methodologies. The determined amounts were 12.69 mmol g⁻¹ for carboxylic groups, 0.89 mmol g⁻¹ for lactones, and 3.74 mmol g⁻¹ for phenols, highlighting a predominance of –COOH groups on the nanostructure’s surface.

**Fig.S5** The presented graph shows the absorbance spectrum of a graphene oxide (GO), which displays a characteristic profile for this material, with a single prominent peak around 230 nm, attributed to the π→π* transition of aromatic C=C bonds in the graphitic regions. The relatively high absorbance intensity suggests good colloidal dispersion of the GO and a significant concentration of oxidized sheets. The continuous decrease in absorbance across the spectrum, extending into the near-infrared (NIR) region, is associated with n→π* transitions of C=O groups and scattering effects from partially reduced or aggregated sheets. This spectral behavior indicates the presence of oxygen-containing functional groups, suggesting the material is still oxidized, though partially reduced with some conjugated domains. However, the absence of a new peak near 300 nm confirms that the material remains predominantly in its oxidized form (GO), rather than being reduced graphene oxide (rGO) (Serodre et al., 2019; Marcano et al., 2010).

**Fig. S6** X-ray diffraction (XRD) was used to characterize the conversion of natural graphite (expanded and milled) into graphite oxide (GrO). The obtained diffractogram shows a peak around 10°, corresponding to the (002) reflection of graphite oxide, and the absence of the peak at 26.5°, which is typical of the (002) reflection of natural graphite (expanded and milled), indicating the effective conversion of graphite into GrO (Pisapia et al., 2009; Stobinski et al., 2014; Saleem et al., 2018). Based on Bragg’s Law, an interlayer spacing of 8.0 Å was calculated for GrO. This increase compared to the spacing of expanded graphite (3.4 Å) is attributed to steric and electrostatic repulsion effects caused by the oxygen-containing groups introduced onto the surface of the layers, which weaken the π–π interactions between the graphitic planes.

**References**

Botella, R., Piñeiro-García, A., Semetey, V., Lefèvre, G. (2022). Polarized ATR-IR spectroscopy for the identification of material structure: The case of graphene oxide. Mater. Lett, *320*, 132352. <https://doi.org/10.1016/j.matlet.2022.132352>

Cançado, L. G., Jorio, A., & Pimenta, M. A. (2007). Measuring the absolute Raman cross section of nanographites as a function of laser energy and crystallite size. Physical Review B, 76(6), 064304. <https://doi.org/10.1103/PhysRevB.76.064304>

Jorio, A., Saito, R., Dresselhaus, G., Dresselhaus, M.S. (2011) Raman spectroscopy in graphene related systems. John Wiley & Sons.

Konkena, B., Vasudevan, S. (2012). Understanding Aqueous Dispersibility of Graphene Oxide and Reduced Graphene Oxide through pKa Measurements. J. Phys. Chem. Lett*.*, *3*,867–872. <https://doi.org/10.1021/jz300236w>

Marcano, D. C., Kosynkin, D. V., Berlin, J. M., Sinitskii, A., Sun, Z., Slesarev, A., Alemany, L. B., Lu, W., & Tour, J. M. (2010). Improved Synthesis of Graphene Oxide. ACS Nano, 4(8), 4806–4814. <https://doi.org/10.1021/nn1006368>

Pisapia, D., Barrios, C., Martínez, M., Reategui, K.,Meléndez, W., Marrero, S., Escobar, M. (2009). Oxidation of natural graphite in the laboratory and comparison with the synthetic graphite oxide, by means of thermal and spectroscopic techniques. Rev. Téc. Ing. Univ. Zulia, 32, 3, 249-255**.**

Saleem, H., Haneef, M., & Abbasi, H. Y. (2018). Synthesis route of reduced graphene oxide via thermal reduction of chemically exfoliated graphene oxide. *Materials* Chemistry and Physics, 204, 1–7. <https://doi.org/10.1016/j.matchemphys.2017.10.020>

Serodre,T. , Oliveira, N. A. P., Miquita, D. R., Ferreira, Max P., Santos, A. P., Resende, V.G., Furtado, C. A. (2019). Surface silanization of graphene oxide under mild reaction conditions. J. Braz. Chem. Soc., 30, 11, 2488-2499. <http://dx.doi.org/10.21577/0103-5053.20190167>.

Stobinski, L., Lesiak, B., Malolepszy, A., Mazurkiewicz, M., Mierzwa, B., Zemek, J., Jiricek, P., & Bieloshapka, I. (2014). Graphene oxide and reduced graphene oxide studied by the XRD, TEM and electron spectroscopy methods. Journal of Electron Spectroscopy and Related Phenomena, 195, 145–154. <https://doi.org/10.1016/j.elspec.2014.07.003>

Surekha, G., Krishnaiah, K. V., Ravi, N., Padma Suvarna, R. (2020). FTIR, Raman and XRD analysis of graphene oxide films prepared by modified Hummers method.  J. Phys.: Conf. Ser.,1495, 012012. <https://doi.org/10.1088/1742-6596/1495/1/012012>

Liu, Z., Robinson, J. T., Sun, X., Dai, H. (2008). PEGylated Nanographene Oxide for Delivery of Water-Insoluble Cancer Drugs. J. Am. Chem. *Soc.*, *130*(33), 10876–10877. <https://doi.org/10.1021/ja803688x>.
